# Poster Session II - A293 DEVELOPMENT OF A MACHINE LEARNING MODEL FOR THE USE OF BLOOD BIOMARKERS TO PREDICT IBD PRESENCE AND ACTIVITY

**DOI:** 10.1093/jcag/gwaf042.293

**Published:** 2026-02-13

**Authors:** K R Fine, D Mulder, E Lehman, P Briand, J Britton

**Affiliations:** Medicine, Queen’s University, Kingston, ON, Canada; Medicine, Queen’s University, Kingston, ON, Canada; Medicine, Queen’s University, Kingston, ON, Canada; Medicine, Queen’s University, Kingston, ON, Canada; Medicine, Queen’s University, Kingston, ON, Canada

## Abstract

**Background:**

Inflammatory bowel disease (IBD) is a lifelong condition involving a complex interaction of immune mediators. The current gold standard evaluation includes invasive testing like colonoscopies. Underserved communities can face significant barriers to accessing colonoscopies and other common imaging procedures. Remote rural and indigenous communities can be far from the nearest hospital that possesses imaging equipment, requiring patients of all ages be transported outside their own communities. While IBD is multivariate, it is known that white blood cells (WBCs) and inflammation-related proteins play a role in IBD pathology, and the levels of these biomarkers can be measured using a blood test. The effects of each individual biomarker and their interactions between different disease states can be tracked and analyzed using machine learning (ML), and by applying the results of this analysis to new patient data it is possible to predict the IBD activity status of these new patients using a simple blood test that can be performed cheaply and in local health centers.

**Aims:**

Create an ML model that uses inflammation-associated biomarker levels to differentiate between healthy patients, those with active IBD, and those with IBD in remission, as while as between patients with Inflamatory Bowel Disease and active IBD and diagnose patients into these classes.

**Methods:**

1458 bloodwork measurements from 108 IBD patients were included in this analysis. Disease activity was classified by physician global assessment score. A variety of ML and deep learning (DL) models, including random forest, support vector machine, multilayer perceptron, and keras were trained and evaluated for diagnosis and classification.

**Results:**

The optimal model, random forest, achieved an accuracy of 73% and a specificity of 87% when distinguishing between healthy, active IBD, and remission IBD samples. It achevied an accuracy of 80% and a specificity of 88% when distinguishing between IBS and active IBD samples. The features with the highest relative importance to the model were platelets, hemoglobin, and neutrophils.

**Conclusions:**

An ML model can be created to diagnose and track IBD efficiently and non-invasively, presenting a promising proof-of-principle for the ML-facilitated use of routine bloodwork for the diagnosis of complex immunologic conditions. These findings, once validated, could save unnecessary invasive testing and health care costs.

A293 Table 1: Model accuracy differentiating control, active, and remission samples

Funding Agencies: Queen’s University and Kingston General Hospital

**Table:** 

Model	accuracy	F1 statistic	Specificity	Sensitivity
Random Forest	0.72	0.71	0.86	0.72
XGBoost	0.67	0.67	0.84	0.67
SVM	0.68	0.67	0.84	0.67
MLP	0.61	0.61	0.81	0.62
MLP with brulee engine	0.64	0.64	0.83	0.66
Keras	0.61	0.52	-	-

